# Molecular characterization of methicillin-resistant *Staphylococcus aureus* strains by *spa* typing and pulsed field gel electrophoresis methods

**DOI:** 10.1186/s12866-018-1305-6

**Published:** 2018-10-24

**Authors:** Tülin Güven Gökmen, Yıldız Kalayci, Akgün Yaman, Fatih Köksal

**Affiliations:** 1 Ceyhan Veterinary Faculty, Department of Microbiology, Cukurova University, 01930 Adana, Turkey; 2 Microbiology Laboratory, Adana City Hospital, 01380 Adana, Turkey; 3 Department of Microbiology, Medical Faculty, Cukurova University, 01380 Adana, Turkey; 4 Present address: Microbiology Laboratory, Adana City Hospital, 01380 Adana, Turkey

**Keywords:** *spa* typing, PFGE, MRSA, Protein a, Epidemiology

## Abstract

**Background:**

Rapid detection of sources and transmission routes by molecular methods provides key data for risk management of methicillin-resistant *Staphylococcus aureus*-induced infections acquired in both the community and hospitals. This study aimed to determine the clonal relationship of methicillin-resistant *S. aureus* strains isolated from our hospital by pulsed-field gel electrophoresis (PFGE) and Staphylococcal protein A (*spa*) typing methods and to identify the predominant clones in Cukurova Region, Turkey.

**Results:**

All isolates analyzed by PFGE were distributed among 11 clusters. Clusters A (*n* = 19) and B (*n* = 27) were 84.1% similar and accounted for 61% of all samples. All isolates were distributed among 18 *spa* types, with the most common type being t030 with 31 isolates (41.3%), followed by t223 with nine isolates (12%) and t127 with seven isolates (9.3%).

**Conclusions:**

We found that t030 was the most common *spa* type in the area where the study was conducted, as also previously shown in studies undertaken in Turkey. However, the rate of t030 in our study was below the rates reported in the literature. We also detected some rare or sporadic *spa* types like t127, which has not been previously defined in our country. We consider that the *spa* typing and PFGE methods are useful for research on clonal relations in monitoring the changing prevalent clones in specific regions.

## Background

*Staphylococcus aureus,* which can be found as floral bacteria in the skin and mucous membranes of humans, have the ability to develop resistance in a short time to broad-spectrum antibiotics, such as the β-lactam group of antibiotics, aminoglycosides, and quinolones that are widely used for the treatment of severe infections in clinical practice [1]. Methicillin-resistant *S. aureus,* first reported in the UK in 1961, remains to be a serious problem for hospitals. Various hospital-acquired methicillin-resistant *S. aureus* clones have spread across the world, becoming a major cause of mortality and morbidity in hospitals. The community-acquired MRSA clones first appeared at the beginning of the 1990s; then, some successful clones spread to hospitals and became predominant in the USA and Europe [2].

In order to identify the epidemiologic characteristics of MRSA strains and more importantly to study the evolution and spreading of epidemic clones, there is a need to employ applicable and reproducibility molecular methods with sufficient discriminative power that are capable of monitoring changes in time. The methods mostly used for this purpose include pulsed-field gel electrophoresis (PFGE), Multilocus Sequence Typing (MLST), staphylococcal cassette chromosome mec (SCC*mec*) typing, and staphylococcal protein A (*spa)* typing [3]. It is agreed that the PFGE method is the gold standard particularly for the short-term surveillance of *S. aureus* typing [3, 4]. Despite the difficulties in reproducibility and interlaboratory reliability, many countries have established a nomenclature for their local pulsotypes through the standardization of PFGE protocols [5, 6]. Sequence-based methods, such as MLST and *spa* typing are highly reproducible among laboratories, can easily be standardized, and have common names worldwide, rendering these methods more advantageous compared to the PFGE method [6, 7]. Although MLST has a lower distinction power than *spa* typing, it is a superior method for monitoring clonal evolution. The *spa* typing method, on the other hand, not only provides an sufficient discriminative power but also has the advantage of being cost-effective as it targets a single locus [8]. Moreover, the analysis of *spa* types using the based upon repeat patterns (BURP) algorithm has been found highly comparable to MLST [9].

This study aimed to detected the clonal relationship of MRSA strains isolated from clinical samples in our hospital by pulsed-field gel electrophoresis (PFGE) and Staphylococcal protein A (*spa*) typing methods and to determinated the predominant clones in Cukurova Region, Turkey.

## Methods

A total of 197 non-duplicate *Staphylococcus aureus* (*S.aureus*) in 311 *Staphylocoocus spp.* isolates from different patients were collected between November-2012 and December-2013 from the clinical samples sent to the Central Laboratory of Çukurova University, Balcali Hospital, Turkey (Tables 1 and 2).

**Table 1:** Study Patients Characteristics and Epidemiologic Classification

Characteristics	*n* = 75	percent
Sex: F/M	23/52	30.6/69.4
Age Distribution:		
Median/IQR	34/0–85	–
Inpatients/Outpatients	64/11	85.3/14.7
Samples		
Wound IP/OP	20/10	26.7/13.3
Tracheal aspirate IP/OP	31/−	41.3/−
Blood IP/OP	11/−	14.7/−
Urine IP/OP	2/1	2.7/1.3

*F* Female, *M* Male, *IQR* Interquartile range, *IP* Inpatients, *OP* Outpatients

**Table 2:** Distribution of isolates to hospital units

Unit	Isolates number (%)
Pediatric Intensive Care Unit	10 (13%)
Reanimation Intensive Care Unit	10 (13%)
Internal Medicine Intensive Care Unit	5 (6.7%)
Chest Disease Unit	4 (5.3%)
Infectious Disease Unit	4 (5.3%)
Dermatology Unit	4 (5.3%)
Burn Unit	3 (4%)
Pediatric Oncology Unit	2 (2.7%)
Pediatric Haematology Unit	2 (2.7%)
Pediatric İnfectious Disease Unit	2 (2.7%)
Pediatric 2 Unit	2 (2.7%)
Nephrology Unit	2 (2.7%)
Urology Unit	2 (2.7%)
Neurology Intensive Care Unit	2 (2.7%)
Endocrinology Unit	2 (2.7%)
Plastic and Reconstructive Surgery Unit	2 (2.7%)
Haematology Unit	2 (2.7%)
Neonatal Intensive Care Unit	1 (1.3%)
Pedatric Allergy and Immunology Unit	1 (1.3%)
Neurosurgery Unit	1 (1.3%)
Ortopedics and Traumatology Unit	1 (1.3%)
Gynecology and obstetrics unit	1 (1.3%)
General Surgery Unit	1 (1.3%)
Pediatric İnfectious Disease Unit 2	1 (1.3%)
Oncology Unit	1 (1.3%)
Ophtalmology Unit	1 (1.3%)
Pediatric Gastroenterology Unit	1 (1.3%)
Pediatric Surgery Unit	1 (1.3%)
Neurology Unit	1 (1.3%)
Urology Unit 2	1 (1.3%)
Neurosurgery Unit	1 (1.3%)
Pediatrics Cardiovascular surgery Unit	1 (1.3%)
Total	75

The identification and antibiotic sensitivity tests of the strains were performed on the Vitek 2 automated system for oxacillin, gentamicin, ciprofloxacin, erythromycin, clindamycin, linezolid, teicoplanin, vancomycin, imipenem, tetracycline, tigecycline, fosfomycin, fusidic acid, rifampicin and trimethoprim/sulfamethoxazole. Confirmation of 75 isolates with methicillin resistance was confirmed by the *Kirby*-*Bauer disc* diffusion method for cefoxitin. Breakpoints were applied according to the 2016 European Committee on Antimicrobial Susceptibility Testing (EUCAST) guidelines [10].

The SmaI-PFGE was performed as described in a previous study [11]. The band profiles were analyzed using GelCompar II software (version 4.0; Applied Maths, Sint-Martens-Latem, Belgium).

Deoxyribonucleic acid (DNA) was extracted from isolates by mechanical lysis using a Mickle tissue disintegrator (Mickle Laboratory Engineering Co. Ltd.). Polymerase chain reaction (PCR) was applied to DNA extracts using the *spa*-1113f (5’TAA AGA CGA TCC TTC GGT GAG C3’) and *spa*-1514r (5’CAG CAG TAG TGC CGT TTG CTT3’) primers [12]. The PCR products were purified using SentroPure® DNA purification kit (Sentromer DNA Technologies LLC, Istanbul, Turkey) according to the kit protocol. Then, cycle sequencing was undertaken using BigDye® Terminator v3.1 cycle sequencing kit (Applied Biosystems, CA, USA). The products were purified with ZR DNA Sequencing Clean-up Kit™ (Zymo Research, CA, USA) as recommended in the protocol. The DNA sequence analysis of the *spa* gene region was performed using ABI Prism 310 DNA sequencer (Applied Biosystems, CA, USA).

The raw data was processed with Sequencing Analysis Software version 5.1, and the *spa* types were identified by Ridom StaphType TM (Ridom GmbH, Würzburg, Germany) software. Using the BURP algorithm in the software, *spa* types were grouped based on six or less repeat differences.

## Results

In this study, we determined antibiotic resistance of 75 MRSA isolates by the Vitek 2 automated system routinely used in the laboratory (Table 3).

**Table 3:** Antibiotic resistance of MRSA isolates

Antibiotics	CIP	DA	E	FOS	FA	CN	LZD	MOX	RA	TEI	TE	TIG	SXT	VA
Resistant strains number	39	43	46	26	5	36	–	32	39	–	47	–	13	–
%	52	57.3	61.3	34.6	6.7	48	0	42.7	52	0	62.6	0	17.3	0

*CIP* Ciprofloxacin, *DA* Clindamycin, *E* Erythromycin, Fusidic acid, *FOS* Fosfomycin, *CN* Gentamycin, *IPM* Imipenem, *OX* Oxacillin, *LZD* Linezolid, *RA* Rifampin, *TEI* Teicoplanin *TE* Tetracycline, *TIG* Tigecycline, *SXT* Trimethoprim-sulfametaxazole, *VA* Vancomycin

All of the isolates were evaluated with PFGE and *spa* typing to reveal their epidemiology in our region. When the similarity cut-off value was taken as 85%, 75 isolates were divided into 11 main clusters by the PFGE method. The largest cluster was cluster B with 27 members, followed by cluster A with 19 members. Clusters J, K, L and N had a single isolate. Clusters A and B constituted 61.3% of all isolates with a similarity of 84.1% according to PFGE.

The most common *spa* type was t30 with 31 isolates (41.3%), followed by t223 with nine isolates (12%) and t127 (9.3%) with seven isolates. Eight of the isolates were singletons (Table 4).

**Table 4:** Distribution of test isolates by their *spa* types according to samples and spa types

Spa types	PFGE types	Outpatients				Inpatients				Samples Number (%)
		TA	Wound	Blood	Urine	TA	Wound	Blood	Urine	
t030	A	–	–	–	–	11	3	5	1	31 (41.3%)
	B	–	–	–	1	8	1	–	–	
	C	–	–	–			1			
t223	G	–	–	–	–	1	3	1	1	9 (12%)
	H	–	–	–	–	–	–	2	–	
	J	–	–	–	–	1	–	–	–	
t127	B	–	2	–	–	3	–	–	–	7 (9.3%)
	F	–	–	–	–	2	–	–	–	
t037	B	–	–	–	–	–	1	–	–	6 (8%)
	D	–	–	–	–	–	1	2	–	
	G	–	–	–	–	–	1	1	–	
t005	G	–	2	–	–	–	1	–	–	4 (5. 3%)
	M	–	1	–	–	–	–	–	–	
t002	B	–	1	–	–	–	1	–	–	2 (2.7%)
t267	G	–	1	–	–	–	–	–	–	2 (2.7%)
	N	–	–	–	–	–	1	–	–	
t008	B	–	–	–	–	–	2	–	–	2 (2.7%)
t016	B	–	–	–	–	–	2	–	–	2 (2.7%)
t790	B	–	–	–	–	1	1	–	–	2 (2.7%)
t304	C	–	1	–	–	–	–	–	–	1 (1.3%)
t459	D	–	–	–	–	1	–	–	–	1 (1.3%)
t359	K	–	–	–	–	–	1	–	–	1 (1.3%)
t311	A	–	–	–	–	–	1	–	–	1 (1.3%)
t091	G	–	1	–	–	–	–	–	–	1 (1.3%)
t7576	A	–	–	–	–	1	–	–	–	1 (1.3%)
t2816	N		1	–	–	–	–	–	–	1 (1.3%)
t4565	M	–	–	–	–	–	1	–	–	1 (1.3%)
Total		11				64				75 (100%)

*TA* Tracheal aspirate

All the t030 isolates were in clusters A and B, except for one isolate in cluster C. Clusters A and B contained nine more *spa* types. The strains in cluster G, the third largest cluster, were distributed among five different *spa* types.t223 was the second most prevalent *spa* type and was distributed among clusters G, H and J, showing 80.1% similarity in SmaI-PFGE. The third common *spa* type, t127, was included in cluster B (Table 5).

**Table 5:**
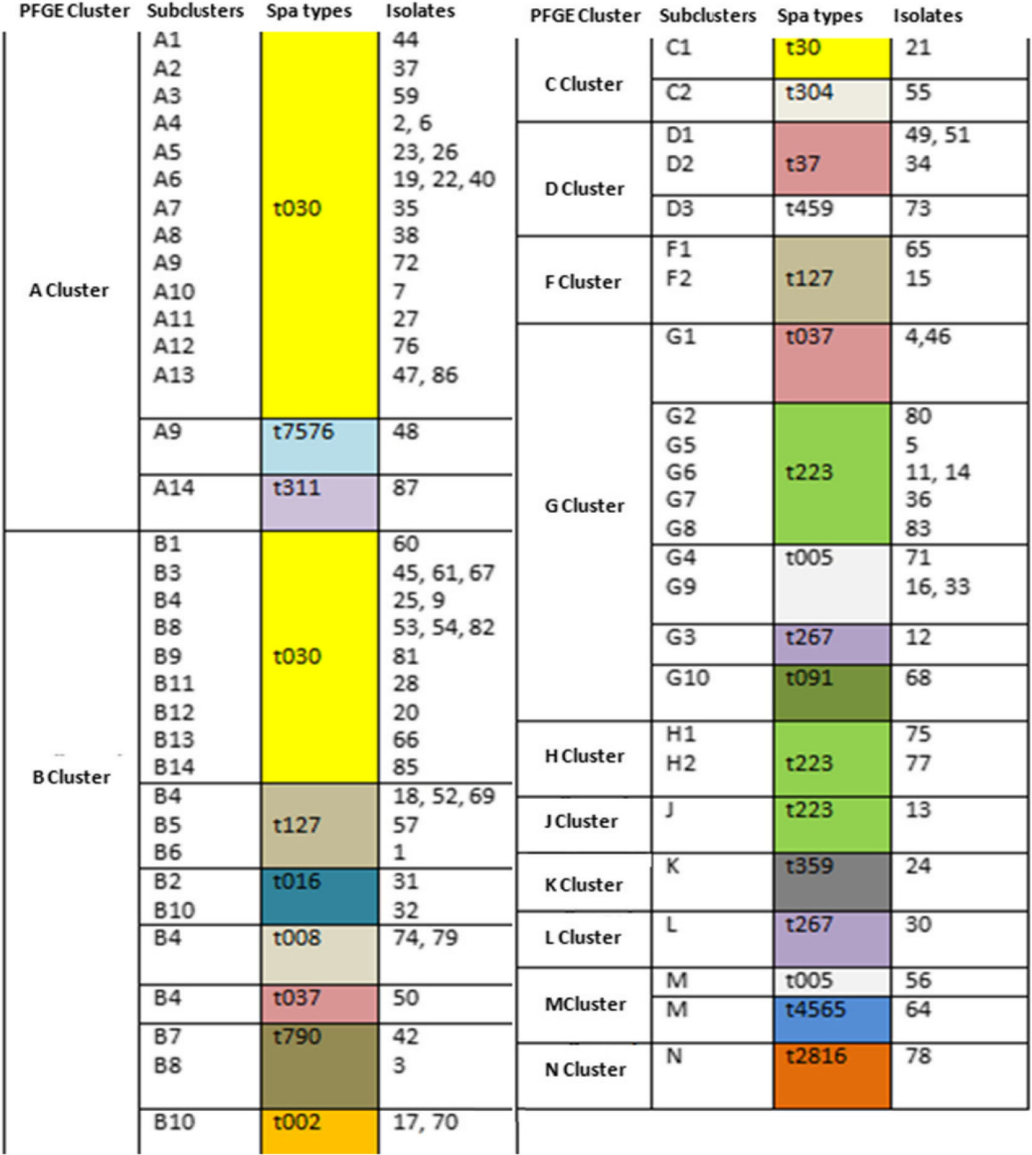
Comparison of *S.aureus* strains with PFGE and spa typing

**Fig. 1:**
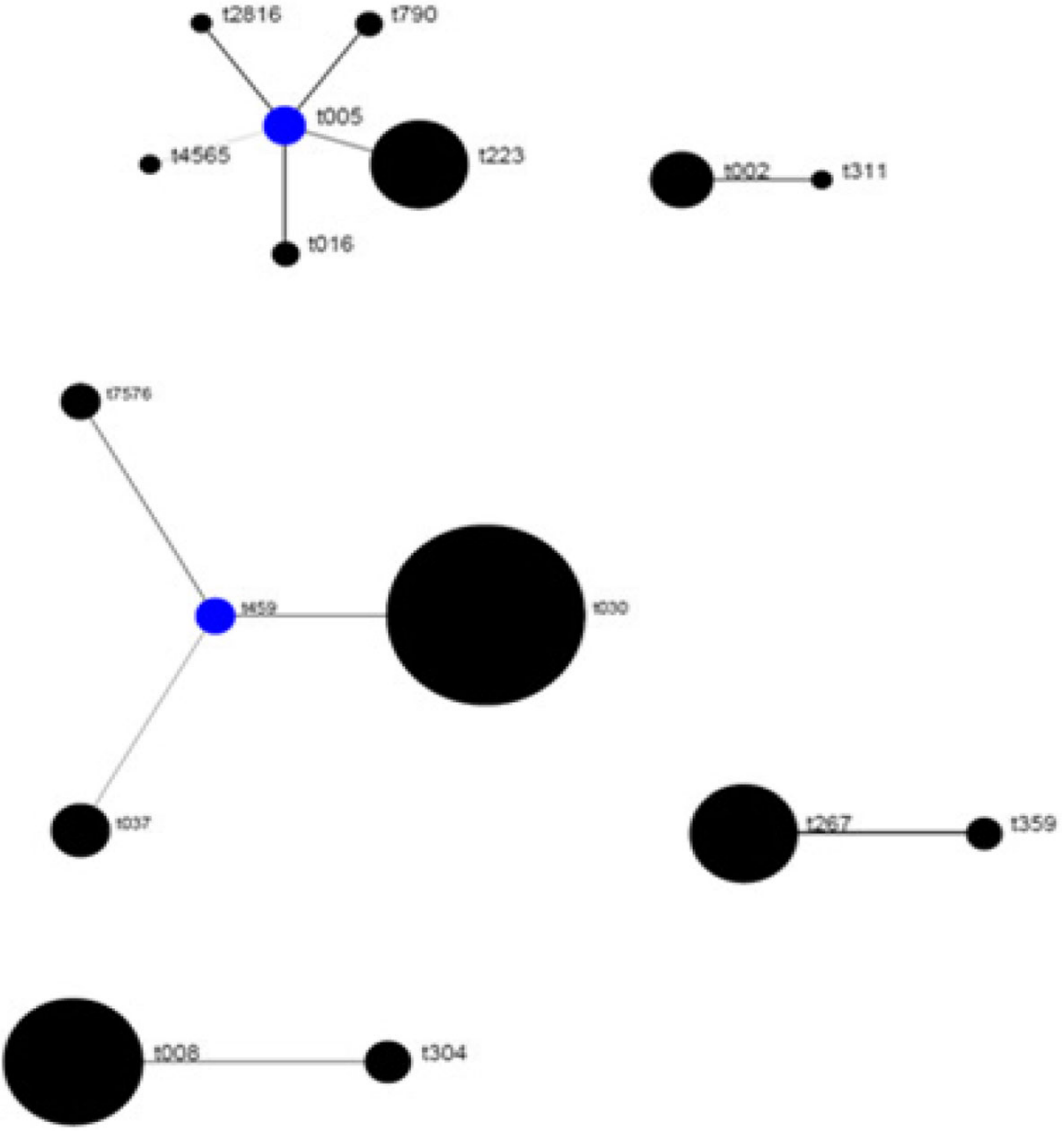
Distribution of spa clonal complexes (BURP analysis)

The *spa* types were divided into five clusters by the BURP analysis. Cluster 1 [Clonal Complex 005 (CC005)] consisted of t790, t223, t4565, t2816 and t016 originating from t005. Cluster 2 (CC459) contained t37, t30 and t7576 originating from t459 (Fig. 1).

## Discussion

Although many MRSA genotypes and clonal clusters have been identified in different biogeographic regions, it has been generally determined that certain gene clones are predominant. In the United States, community-associated ST8-spa t008 (USA300) and ST5-spa t002 clones are predominant, while ST22-spa t032, mostly associated with hospital, is more commonly seen [6, 13–15]. In France and Belgium, however, it is reported that the ST8-spa t008 clone, defined as the community origin similar to the USA, is widespread [14, 15]. In Bulgaria and the Balkan countries, such as Romania and Turkey, ST239-spa t030 is the predominant *spa* type [14]. It has been shown in studies conducted in Asia and Far East countries that ST239-spa t030, ST239-spa t037 and ST5-spa t002 are the most common types [16–18].

In the current study, 75 MRSA clinical isolates were examined. Twenty-eight isolates (37.3% of all isolates) were collected from intensive care units where MRSA infections were most prevalent [19]. Clonal relationship of MRSA strains were determined by pulsed-field gel electrophoresis (PFGE) and Staphylococcal protein A (*spa*) typing methods. In many studies similar to our study, MLST method was used together with spa typing and PFGE methods. However, MLST studies of these isolates has to be postpone due to funding reasons.

The isolates were distributed among 11 clusters with PFGE and 18 clusters with *spa* typing. *Spa* typing revealed that 31 isolates (41.3%) belonged to type t030. The other *spa* types detected were t223 (12%) with nine isolates, t127 (9.3%) with seven isolates, t037 (8%) with six isolates, and t005 (5.3%) with four isolates. This finding indicates that type t030 has high prevalence (41.3%) in Turkey, as in Asian and Balkan countries [14, 17].

t030 isolates were collected in clusters A and B, which were 80% similar to each other according to the PFGE band profiling. The second most frequent *spa* type, t223, was found in clusters G, H and J, which were 80.1% similar as revealed by PFGE. The third *spa* type, t127, was included in cluster B. Rare or sporadic *spa* types showed a heterogeneous distribution with PFGE. In a Finnish study, 90% of the most common *spa* types, t172 and t067, were clustered in FIN-4 and FIN-16 PFGE patterns, respectively. Similar to our study, rare *spa* types were associated with more than one PFGE cluster [20].

The ST239 clone containing spa t030 was first found to be widely distributed in a large number of patients in Brazil; therefore, this clone was called the Brazilian clone. Later spreading to the neighboring South American countries, such as Argentina, Chile, Uruguay, and European countries, including Portugal, Czech Republic, and Greece, the ST239 clone is now epidemically seen in most Asian countries [17, 21].

In a study conducted in China, it was reported that the predominant clone from 1994 to 2000 was ST239-spa t037 with 92.6% prevalence, but after 2000, this clone rapidly decreased and replaced by ST239-spa t030. In this study, *spa* types t30 and t37 were located in unrelated clusters by PFGE [17].

In Turkey, the first spa t030 isolates were identified from nine blood cultures in 2003–2004. Then, in a study conducted with 54 strains isolated from invasive infections (92% blood cultures) in eight university hospitals between 2005 and 2006, 89% of the strains were identified as spa t030. The rate of spa t030 in the current study was below the values given in these two studies. We consider that this difference may be due to the variances in the samples.

In another study, 48 MRSA isolates were divided into 14 PFGE clusters and the *spa* type of all isolates except two was spa t030 which collected in 3 months. As noted in the study, because the samples reflected a period of 3 months, genetic diversity may be limited [22–24].

However, our study, which shows that some community-based isolates are beginning to be seen in hospitals at increasing frequency, will be a guide for future studies.

In a 2008–2009 study conducted in 12 cities in Turkey, the most common PFGE pattern was found to be Pulsotype A (91.4%) and the most common *spa* type was t030 (85.1%) among the 397 MRSA isolates collected. This clone was called ‘TR09’. However, in the same study, it was determined that among the 91 isolates obtained in 2011, the rate of t30 decreased to 70.3% and the number of new sporadic types increased. The reduction in the rate of spa t030 in this study supported our work. This study showed that the most common community-associated MRSA clone was ST737-spa t005, which was named “TR10” [25]. We also found the prevalence of the t005 type as 5.3%. In addition, we also detected t790, t4565, t2816, and the second most common spa type t223 in our study, clustered in the same clonal complex with t005 by BURP analysis.

In our study, we identified t127 as the third most commonly defined *spa* type, which had not been previously defined in Turkey. Methicillin-sensitive and resistant t127 clones have been isolated from humans, food and animals in various studies and are considered community-associated [26–28].

## Conclusion

In conclusion, among the MRSA strains, spa t030 was predominant in our region, as previously reported for other regions in Turkey. The most common *spa* types were closely clustered in PFGE. In the current study, spa t127, which is common among community-acquired isolates, was identified for the first time in Turkey. Therefore, it is considered that MRSA surveillance is absolutely required to constantly monitor clones across the world. Furthermore, it is beneficial to use the *spa* typing method and PFGE in the research of clonal relations to follow the changing clones that are prevalent in Turkey.

## Abbreviations

MRSA: Methicillin-Resistant *Staphylococus aureus*
PFGE: Pulsed-Field Gel Electrophoresis
*spa*: Staphylococcal protein A
